# TUXEDO: a phase I/II trial of cetuximab with chemoradiotherapy in muscle‐invasive bladder cancer

**DOI:** 10.1111/bju.15864

**Published:** 2022-08-16

**Authors:** Nicholas D. James, Wenyu Liu, Sarah Pirrie, Baljit Kaur, Carey Hendron, Daniel Ford, Anjali Zarkar, Richard Viney, Elizabeth Southgate, Amisha Desai, Syed A. Hussain, the TUXEDO investigators, Jim Barber, Isabel Syndikus, Zafar Malik, Chinnamani Eswar, Stephen Mangar, Julian Money‐Kyrle, Anna Lydon, Johannes Van Der Voet, Nicholas James, Anjali Zarkar, Dan Ford

**Affiliations:** ^1^ Institute of Cancer Research London UK; ^2^ Cancer Research UK Clinical Trials Unit (CRCTU) University of Birmingham Birmingham UK; ^3^ Cancer Centre University Hospitals Birmingham NHS Foundation Trust Birmingham UK; ^4^ Department of Oncology & Metabolism The Medical School Sheffield UK

**Keywords:** muscle‐invasive bladder cancer, cetuximab, clinical trial, chemoradiotherapy, feasibility, #BladderCancer, #blcsm, #uroonc

## Abstract

**Objective:**

To assess the feasibility and preliminary efficacy of adding cetuximab to standard chemoradiotherapy for muscle‐invasive bladder cancer.

**Patients and Methods:**

TUXEDO was a prospective, single‐arm, open‐label, phase I/II trial conducted in six UK hospitals. Cetuximab was administered with an initial loading dose of 400 mg/m^2^ on Day 1 of Week −1, and then seven weekly doses of 250 mg/m^2^. The radiotherapy schedule was 64 Gy/32F with Day 1 mitomycin C (12 g/m^2^) and 5‐fluorouracil (500 mg/m^2^/day) over Days 1–5 and Days 22–26. Patients with T2‐4aN0M0 urothelial cancer and a performance status of 0–1 were eligible. Prior neoadjuvant therapy was permitted. The Phase I primary outcome was impact on radiotherapy treatment completion and toxicity experienced during treatment. The Phase II primary outcome was local control at 3 months post treatment.

**Results:**

Between September 2012 and October 2016, 33 patients were recruited; seven in Phase I, 26 in Phase II. Three patients in Phase II were subsequently deemed ineligible and received no trial therapy. Eight patients discontinued cetuximab due to adverse effects. The patients’ median (range) age was 70.1 (60.6–75.1) years, 20 had a performance status of 0, 27 were male and 26 had already received neoadjuvant chemotherapy. In Phase I, all patients completed planned radiotherapy, with no delays or dose reductions. Of the 30 evaluable patients in Phase II, 25 had confirmed local control 3 months after treatment (77%, 95% confidence interval 58–90). During the trial there were 18 serious adverse events. The study was halted due to slow accrual.

**Conclusion:**

Phase I data demonstrate it is feasible and safe to add cetuximab to chemoradiotherapy. Exploratory analysis of Phase II data provides evidence to consider further clinical evaluation of cetuximab in this setting.

## Introduction

In the UK, approximately 10 200 new cases of bladder cancer are diagnosed each year [1], with approximately 4000 cases of muscle‐invasive bladder cancer (MIBC) each year and 5‐year survival rates of approximately 45% [2, 3].

Selective bladder preservation or radical cystectomy are both options for MIBC [4], with or without neoadjuvant chemotherapy [5]. There are indications from other primary cancer sites that synchronous chemoradiotherapy may produce local control and survival advantages over radiotherapy alone [6]; only two studies have compared this approach in bladder cancer. The first compared radiotherapy with or without cisplatin, demonstrating improved locoregional control [7]. The second, BC2001, demonstrated substantially improved pelvic control rates, with good tolerability for chemo‐radiation using a mitomycin C (MMC)/5‐fluorouracil (5FU) regimen [8, 9, 10]. Long‐term patient‐reported outcomes also show good bladder function in the majority of patients [9].

In BC2001, the 2‐year cystectomy rate was 11.7% (95% confidence interval (CI) 7.3–18.4%), with few invasive recurrences occurring beyond 2 years; therefore, improving bladder preservation would lead to an improved quality of life (QoL) for many patients, as suggested in a recent review [11].

Expression of epidermal growth factor receptor (EGFR) in MIBC correlates with poor prognosis [12]. Cetuximab, a chimeric monoclonal IgG1 antibody directed against the EGFR, blocks binding of endogenous EGFR ligands, thus inhibiting function of the receptor, induces internalization of EGFR, and targets cytotoxic immune effector cells towards EGFR‐expressing tumour cells via antibody‐dependent cellular cytotoxicity [13]. Combining radiotherapy with cetuximab has been shown to improve outcomes compared to radiotherapy alone in head and neck cancer, with minimal additional toxicity [14].

Approximately 50% of MIBC patients in the UK receive radical radiotherapy as their main treatment, which is approximately 2000 patients per year [15]. In addition, pre‐clinical studies support the use of cetuximab in bladder cancer [16, 17, 18, 19, 20, 21]. Therefore, the aims of the TUXEDO trial were to assess the feasibility and preliminary efficacy of adding cetuximab to standard of care chemoradiotherapy in patients with MIBC.

## Patients and Methods

### Study Design

TUXEDO was a single‐arm, non‐randomized, non‐blinded Phase I/II clinical trial, recruiting patients from six hospitals in the UK. Ethical approval for the trial protocol was obtained from the London Bloomsbury Research Ethics Committee and local institutional review boards.

TUXEDO used a modified Phase I/II design to allow assessment of feasibility with two chemoradiotherapy regimens in combination with cetuximab (one cohort per regimen). Feasibility of cetuximab administration was first assessed with MMC/5FU as evaluated in the BC2001 trial. If feasible, Phase II would be initiated using this regimen. However, if feasibility (that is, delivery of the core radiotherapy treatment), or the toxicity profile using cetuximab with MMC/5FU was not acceptable in either Phase I or II, then the design allowed for a return to Phase I to assess cetuximab with cisplatin in two escalating doses. Using the maximum tolerated dose of cisplatin identified, Phase II could then be initiated.

### Patients

Patients aged ≥18 years, with histologically proven MIBC, performance status of 0–1, and adequate bone marrow, hepatic and renal function, who were able to receive radical radiotherapy were eligible for this trial. Previous neoadjuvant chemotherapy (up to 3–4 cycles) was permitted. Patients with severe/uncontrolled cardiovascular disease, inflammatory bowel disease, widespread carcinoma *in situ* (CIS) or CIS remote from the muscle‐invasive tumour, or who had untreated hydronephrosis or who had received previous pelvic radiotherapy, as well as pregnant or breast‐feeding women, were excluded. All patients gave written informed consent for the trial and optional substudy. Transurethral resection of bladder tumour (TURBT) was carried out as per local practice at participating centres. There was no requirement for complete TURBT (this is standard practice in the UK for chemoradiation).

### Procedures

Cetuximab was administered via intravenous infusion once weekly, with an initial loading dose of 400 mg/m^2^ on Day 1 of Week −1, and then seven weekly doses of 250 mg/m^2^. Where relevant, the loading dose of cetuximab was administered within 5 weeks of the last neoadjuvant chemotherapy. Delays beyond 5 weeks for recovery from chemotherapy toxicity were permitted. MMC (12 mg/m^2^) was delivered via intravenous bolus on Day 1 of Week 1 only prior to starting radiotherapy, with 5FU (500 mg/m^2^/day) administered as a continuous i.v. on Days 1–5 and Days 22–26. During Weeks 1–7 of study treatment patients were treated with CT‐planned radical radiotherapy, delivering 64 Gy in 32 fractions to the whole bladder. Where possible, patients were treated as outpatients.

Although not used during TUXEDO, the trial had the option to use cisplatin in a separate Phase I cohort of patients as an i.v. infusion (25 mg/m^2^ escalated to 30 mg/m^2^ if toxicity permitted) on Days 1, 8, 15, 22 and 36 of cetuximab treatment per‐radiotherapy.

For registered patients, pre‐treatment evaluation included: a physical examination, chest CT scan, abdominal and pelvis MRI or CT scan, bladder capacity, full blood counts with liver and kidney function assessed at baseline. Adverse events (AEs), defined according to National Cancer Institute Common Terminology Criteria for Adverse Events (CTCAE) v4.03 [22], were recorded at baseline, Day 1 and Week −1, weekly in Weeks 1–7 and at 30 days post treatment. Radiation Therapy Oncology Group (RTOG) toxicity scoring and QoL assessment were performed 30 days, and at 3, 6, 10 and 16 months post treatment.

Response, local and distant progression were assessed via cystoscopy, and cross‐sectional imaging 3, 6, 10 and 16 months post treatment.

TUXEDO also incorporated an optional substudy requesting use of tissue taken at initial surgery, excess tissue removed at staging, tissue taken during follow‐up cystoscopies, and tissue taken if a patient relapses. Collection of urine samples prior to treatment, during radiotherapy, and at 30 days and 3 months post treatment was also included.

### Outcomes

The primary outcome for Phase I was to determine the feasibility and toxicity profile of cetuximab with MMC/5FU and, if necessary, determine the maximum tolerated dose of cisplatin in combination with cetuximab. Feasibility was based on assessment of using the proposed drugs in combination with radical radiotherapy.

The primary objective of Phase II was to assess preliminary evidence of cetuximab efficacy with the chemoradiotherapy treatment selected from Phase I by assessing whether it improved local control of advanced bladder cancer at 3 months post treatment in comparison to historical controls from BC2001 [10].

The secondary objectives for Phase II were to assess: the toxicity profile and number of toxicities associated with the study treatment; delivery of target radiotherapy; the probability of 6‐ and 12‐month loco‐regional progression‐free interval; the cystectomy rate at 1 year; overall survival time; and patient QoL. The QoL questionnaire booklet comprised the European Organization for Research and Treatment of Cancer (EORTC) QLQ‐C30 for cancer [23] and the EORTC QLQ‐BLM30, a 30‐item MIBC‐specific questionnaire [24]. Time to muscle‐invasive and non‐muscle‐invasive progression, although not prespecified, were analysed ad hoc as additional secondary outcomes.

Recurrence was defined as clinical or radiological progression of disease from clinical remission after completion of therapy. Recurrence was defined as either loco‐regional or distant. Loco‐regional recurrence was defined as recurrence in bladder or nodal recurrence within the true pelvis. Local recurrence was classified as non‐invasive (≤pT1 or CIS) or invasive (≥pT2). Regional recurrence was defined as pelvic lymph node recurrence within the true pelvis.

### Statistical Analysis

Six patients were planned to be recruited to the Phase I cohort and, if feasible in terms of delivery of radiotherapy treatment (as assessed by days’ delay to start of radiotherapy and days’ reduction in length of planned radiotherapy) and if toxicity was acceptable, the trial would proceed to Phase II with this combination. This assessment was not based on any formal hypothesis testing.

The Phase II component assessed the proportion of patients with local control at 3 months post treatment. Phase II used a Simon's two‐stage minimax design with these parameters: Π_0_ = 60% (local control rate at 3 months), Π_1_ = 80%, α = 5%, β = 10% (90% power). A sample size of 45 patients (with at least 33 patients with local control at 3 months post treatment) was needed to give a 5% probability of a false‐positive (incorrectly accepting a treatment with a true 3‐month response rate of 60% or less), and a 10% probability of a false‐negative (incorrectly rejecting a treatment with a true 3‐month response rate of 80% or more). Phase II analysis included all patients from Phase I and Phase II who received the treatment of interest.

The modified design of TUXEDO also allowed for the feasibility and toxicity profile of cetuximab with cisplatin to be assessed, should the MMC/5FU combination not be acceptable at either phase. The trial could then proceed to the Phase II setting with the selected cisplatin dose if deemed feasible.

Finally, whether a Phase III trial should be considered was set to observation of at least 33 patients with local control at 3 months post treatment. Should local control rates lie between 60% and 80%, then the decision to proceed to a Phase III trial would be based on secondary outcome measures of toxicity and the QoL substudy.

Analyses were performed using Stata 15.1. An independent Safety Review Committee reviewed interim data annually to ensure patient safety. There were no formal stopping rules. The trial was registered on ISRCTN: 80733590.

### Role of the Funding Source

The trial was sponsored by the University of Birmingham and run by the Cancer Research Clinical Trials Unit (CRCTU) located there. Funding came from Cancer Research UK (CRUK/09/021) and cetuximab was supplied by Merck Serono Ltd. The trial was initiated and conducted independently by the trial investigators. The funders had no role in trial design, data collection, data analysis, data interpretation or writing of the report. The corresponding author had full access to all the data in the trial and had final responsibility for the decision to submit for publication.

## Results

**Fig. 1:**
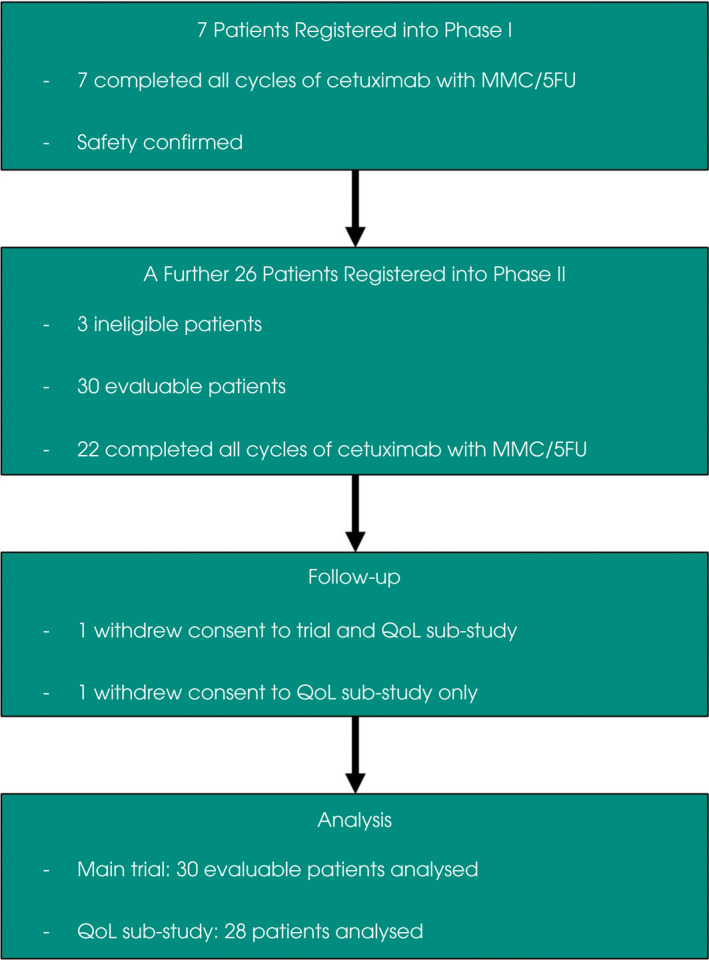
TUXEDO trial profile. Consort diagram of the TUXEDO trial. 5FU, 5‐fluorouracil; MMC, mitomycin C; QoL, quality of life.

Between September 2012 and October 2016, 33 patients were recruited: seven in Phase I and 26 in Phase II (Fig. 1). Three patients in Phase II were subsequently found to be ineligible post registration; they did not receive any study treatment and were non‐evaluable for the outcome measures of the trial. After receiving all trial treatments, one patient withdrew consent for the trial and substudy due to disease progression and lack of mental capacity. In addition, another patient withdrew their consent from the substudy only, but stayed on trial.

The baseline characteristics for patients recruited into Phase I and Phase II are described in Table 1. In total 26/33 patients (78.8%) received up to four cycles of neoadjuvant treatment, the most common regimen being cisplatin and gemcitabine (20 patients).

**Table 1:** Patient characteristics.

	Phase I	Phase II (not evaluable)	Phase II (evaluable)	Total
	*N* = 7	*N* = 3	*N* = 23	*N* = 33
**Age, years**				
Median	69.5	80.9	70.1	70.1
Interquartile range	65.0, 74.7	67.0, 84.8	64.8, 81.0	65.4, 80.2
Range	60.6, 75.1	64.0, 84.8	46.9, 85.6	46.9, 85.6
Age category, *n*/*N* (%)				
<60 years	0/7	0/3	4/23 (17.4)	4/33 (12.1)
60–69 years	4/7	1/3	6/23 (26.1)	11/33 (33.3)
70–79 years	3/7	0/3	5/23 (21.7)	8/33 (24.2)
≥80 years	0/7	2/3	8/23 (34.8)	10/33 (30.3)
**Performance status, *n*/*N* (%)**				
0	3/7	2/3	15/23 (65.2)	20/33 (60.6)
1	4/7	1/3	8/23 (34.8)	13/33 (39.4)
**Sex, *n*/*N* (%)**				
Males	7/7	2/3	18/23 (78.3)	27/33 (81.8)
Females	0/7	1/3	5/23 (21.7)	6/33 (18.2)
**ALT**				
*N*	7	2	23	32
Median, U/L	7.0	10.5	6.0	7.0
Interquartile range, U/L	5.0, 9.0	7.0, 14.0	4.0, 8.0	5.0, 8.0
Range, U/L	5.0, 11.0	7.0, 14.0	2.0, 12.0	2.0, 14.0
**GFR, mL/min/1.73 m** ^**2**^				
*N*	7	2	23	32
Median, mL/min/1.73 m^2^	70.0	76.0	69.0	69.5
Interquartile range, mL/min/1.73 m^2^	69.0, 83.0	68.0, 84.0	46.0, 98.0	56.5, 88.5
Range, mL/min/1.73 m^2^	61.0, 94.0	68.0, 84.0	39.0, 152.0	39.0, 152.0
**Pre‐trial cystoscopy findings, *n*/*N* (%)**				
Biopsy	1/7	0/3	2/23 (9.1)	3/33 (10.0)
Complete tumour resection	3/7	1/3	10/23 (45.5)	14/33 (46.7)
Incomplete tumour resection	1/7	1/3	7/23 (31.8)	9/33 (30.0)
No tumour resection	1/7	0/3	0/23 (0.0)	1/33 (3.3)
Tumour resection completed but extent unknown	0/7	0/3	3/23 (13.6)	3/33 (10.0)
Missing	1	1	1	3
**Pre‐trial neoadjuvant treatment** * **cycles received, *n*/*N* (%)**				
2	0/7	1/3	0/23 (0.0)	1/33 (3.8)
3	2/7	0/3	10/23 (55.6)	12/33 (46.2)
4	5/7	0/3	8/23 (44.4)	13/33 (50.0)
Missing	0	2	5	7
**Primary tumour stage, *n*/*N* (%)**				
T2	4/7	2/3	20/23 (87.0)	26/33 (81.3)
T3a	0/7	0/3	1/23 (4.3)	1/33 (3.1)
T3b	3/7	0/3	0/23 (0.0)	3/33 (9.4)
T4a	0/7	0/3	2/23 (8.7)	2/33 (6.3)
Tx	0	1	0	1
**Primary tumour grade, *n*/*N* (%)**				
G2	0/7	0/3	2/23 (8.7)	2/33 (6.3)
G3	7/7	2/3	21/23 (91.3)	30/33 (93.8)
Missing	0	1	0	1
**Primary tumour size (length)**				
*N*	7	0	17	24
Median, mm^2^	43.0	–	29.0	29.5
Interquartile range, mm^2^	21.0, 50.0	–	17.0, 36.0	18.5, 41.5
Range, mm^2^	10.0, 64.0	–	3.0, 50.0	3.0, 64.0
**Number of tumours found, *n*/*N* (%)**				
1	6/7	1/3	17/23 (89.5)	24/33 (88.9)
2	0/7	1/3	1/23 (5.3)	2/33 (7.4)
3	0/7	0/3	1/23 (5.3)	1/33 (3.7)
Missing	1	1	4	6
**Nodal category, *n*/*N* (%)**				
N0	7/7	1/3	23/23 (100.0)	31/33 (100.0)
Missing	0	2	0	2
**Metastasis**				
M0	7/7	2/3	23/23 (100.0)	31/33 (100.0)
Missing	0	1	0	2
**Histological tumour type, *n*/*N* (%)**				
TCC	6/7	2/3	23/23 (100.0)	31/33 (96.9)
SCC	1/7	0/3	0/23 (0.0)	1/33 (3.1)
Missing	0	1	0	1

ALT, alkaline transferase, GFR, glomerular filtration rate, SCC, squamous cell carcinoma, TCC, transitional cell carcinoma.

* Neoadjuvant treatments consisted of: cisplatin and gemcitabine [20]; cisplatin, gemcitabine, magnesium sulphate and potassium chloride [2]; cisplatin, gemcitabine and nintedanib/placebo [2]; carboplatin and gemcitabine [1]; and cisplatin monotherapy [1].

For the seven patients recruited in Phase I all completed planned radiotherapy of 64 Gy/32F over 44 days; no delays or dose reductions were observed (Table 2). Only one serious AE occurred, leading the study to proceed to Phase II using the MMC/5FU chemotherapy regimen.

**Table 2:** Treatments delivered.

	Phase I patients	Phase II patients	Total patients
	*N* = 7 (%)	*N* = 23 (%)	*N* = 30 (%)
**Radiotherapy treatment**			
Time from registration to radiotherapy, days			
Median	11.0	15.0	13.5
Interquartile range	10.0, 14.0	11.0, 21.0	11.0, 18.0
Range	10.0, 14.0	7.0, 96.0	7.0, 96.0
Duration, days			
Median	44.0	44.0	44.0
Interquartile range	44.0, 44.0	44.0, 45.0	44.0, 44.0
Range	44.0, 44.0	31.0, 50.0	31.0, 50.0
Dose delivered, *n*/*N* (%)			
46 Gy	0/7	1*/23 (4.3)	1/30 (3.3)
64 Gy	7/7	22/23 (95.7)	29/30 (96.7)
Dose fractions, *n*/*N* (%)			
23	0/7	1/23 (4.3)	1/30 (3.3)
32	7/7	22/23 (95.7)	29/30 (96.7)
Inverse‐planned IMRT, *n*/*N* (%)			
No	2/7	5/23 (21.7)	7/30 (23.3)
Yes	5/7	18/23 (78.3)	23/30 (76.7)

	Dose intensity, mg/m^2^/day	Relative dose intensity, %
Cetuximab (planned dose; 268.8 mg/m^2^/day)		
*N*	30	30
Median	269.3	100.2
Interquartile range	266.1, 275.5	99.0, 102.5
Range	177.4, 352.6	66.0, 131.2
**Chemotherapy**		
MMC (planned dose: 12 mg/m^2^)		
*N*	30	30
Median	11.9	99.0
Interquartile range	11.8, 12.0	98.3, 100.4
Range	11.6, 17.9	96.9, 149.0
5FU (planned dose: 500 mg/m^2^/day)		
*N*	30	30
Median	498.9	99.8
Interquartile range	493.8, 506.0	98.8, 101.2
Range	199.1, 929.2	39.8, 185.8

5FU, 5‐fluorouracil; IMRT, intensity‐modulated radiation therapy; MMC, mitomycin C.

* Patient did not complete radiotherapy as planned, having experienced a serious adverse event due to interstitial pneumonitis.

TUXEDO failed to recruit to its prespecified target for Phase II of 45 patients and was halted due to slow accrual. Exploratory analyses of the 30 evaluable patients recruited in Phase II are described. Twenty‐three patients in total were confirmed to have maintained local disease control at 3 months post treatment (77%, 95% CI 58–90%). Of the seven patients who did not maintain local disease control, one died from bladder cancer‐related causes.

All 30 evaluable patients in Phase I and II started radiotherapy treatments. The dose of radiotherapy delivery is summarized in Table 2, as well as the dose intensity and relative dose intensity for cetuximab, MMC and 5FU. Dose delay/modification/omission during the treatment period for cetuximab occurred 24 times in a total of 14 patients. The main causes were administrative reasons (eight patients), toxicity (six patients) and patients feeling unwell (three patients). One patient recruited into Phase II received a higher dose of MMC than defined in the protocol: 17.9 mg/m^2^ per BSA unit. One patient recruited into Phase I received a lower dose of 5FU than defined in the protocol (995.4 mg/m^2^) and another received a higher dose (3735.2 mg/m^2^). In addition, nine patients reported a dose change/delay/interruption to their 5FU treatment, seven of whom did not receive 5FU at Week 4 because of safety concerns. Similar rates of incomplete administration in Week 4 were observed in BC2001 [10].

In total, during the trial, there were 483 AEs, 34 of Grade ≥3, 353 of which were considered to be at least possibly related to trial treatment (Appendix S2). The most common Grade ≥3 AE was diarrhoea (four occurrences). Although no patients withdrew from the trial, eight patients discontinued cetuximab treatment early because of AEs. Despite this, the median delivered dose intensity was 100% (interquartile range 99–102) with the lowest rate of delivery being 66% of target dose; hence, drug exposure levels were consistent and high. One serious adverse reaction was reported in Phase I and 17 were reported in Phase II, two of which were classed as suspected unexpected serious adverse reactions (Table 3).

**Table 3:** Serious adverse reactions.

Phase	SAE category	Event description	Onset, weeks	Duration, weeks	Related treatment
1	SAR	Potassium level dropped	2.9	0.3	C
2	SAR	Feeling unwell, dizzy and diarrhoea (G3)	7.0	0.3	All
2	SAR	Haematuria and acute kidney injury	40.7	33.3	RT
2	SAR	Short of breath, clot on lung and haematuria	11.9	1.0	All
2	SAR	Pyrexia and diarrhoea (G3)	3.1	0.6	C, 5FU, RT
2	SAR	Nausea (G2), anorexia (G2), fatigue (G2) and low sodium levels	8.0	1.0	C, RT
2	SAR	Atrial fibrillation	4.9	1.4	C, MMC/5FU
2	Fatal SUSAR	Shortness of breath and coughing profusely queried neutropenic sepsis but subsequently ruled out	6.4	7.7	C
2	Unrelated SAE	Extensive axillary vein thrombus, fever *Staphylococcus* in blood culture and wound swab from line entry site	6.0	5.0	–
2	SAR	Non‐occlusive right popliteal vein DVT	18.1	6.0	All
2	SAR	Bi‐basal atelectasis and background emphysematous, acute large bilateral pulmonary emboli with equivocal signs of heart strain	20.4	0.0	C
2	SAR	Unable to pass urine possibly due to haematuria/clots	8.0	0.7	RT
2	SAR	Haematuria and urinary retention	9.9	0.9	All
2	SAR	Raised temperature, sore throat and on‐going cough	5.4	0.6	C, MMC/5FU
2	SAR	Raised temperature and rigours. Found to have low haemoglobin	3.7	0.9	C, MMC/5FU
2	Unrelated SAE	Increasingly short of breath with decreased haemoglobin	66.1	0.3	–
2	Non‐fatal SUSAR	Extensive diarrhoea (G3) over 2 weeks	14.0	6.0	All
2	SAR	Neutropenic sepsis. Neutropenia (G3), flu‐like symptoms, cough and dyspnoea	7.0	1.0	C, MMC//5FU

5FU, 5‐fluorouracil; All, all trial treatments (cetuximab, MMC/5FU and radiotherapy); C, cetuximab; DVT, deep vein thrombosis; G, grade; MMC, mitomycin C; RT, radiotherapy; SAE, serious adverse event; SAR, serious adverse reaction; SUSAR, serious unexpected serious adverse reaction.

**Fig. 2:**
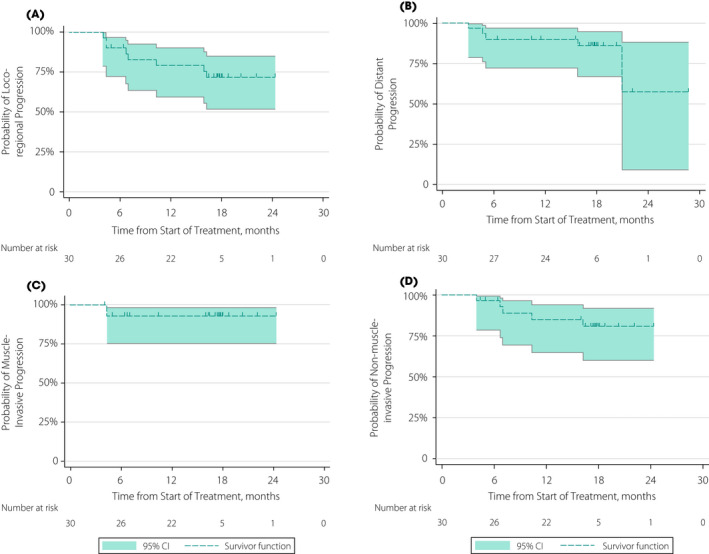
Progression‐free survival. (**A**) Secondary outcome of loco‐regional progression‐free interval probability, defined as the interval in whole days between the start date of treatment and the earliest date of documentation of local recurrence. (**B**) Secondary outcome of distant progression‐free interval probability, defined as the interval in whole days between the start date of treatment to the earliest date of detection of distant recurrence. (**C** and **D**) Secondary outcome of local recurrence as classified by invasive, unequivocal clinical or pathological evidence of muscle wall invasion ≥ pT2, or non‐invasive (≤pT1 including carcinoma *in situ*) recurrence. Patients were censored at date of death or date last seen. Censored patients are indicated by short vertical lines. CI, confidence interval.

For the 30 evaluable patients, the 6‐month loco‐regional progression‐free probability was 90% (95% CI 72–97) and the 12‐month loco‐regional progression‐free probability was 79% (95% CI 59–90; Fig. 2A). Of the eight loco‐regional progressions, six were in the same location as the primary tumour. Distant progression‐free interval probabilities at 6 and 12 months were both 90% (95% CI 72–97; Fig. 2B). Time to muscle‐invasive or non‐invasive progression was also analysed. Six‐ and 12‐month muscle‐invasive progression‐free interval probabilities were both 93% (95% CI 75–98); two patients had Stage T2 muscle‐invasive progression occurring 4 months after start of trial treatment (Fig. 2C). The 6‐ and 12‐month non‐muscle‐invasive progression‐free interval rate was 97% (95% CI 79–99) and 85% (95% CI 65–94), respectively; five patients had progression after the start of trial treatment (Fig. 2D).

One patient underwent cystectomy because of recurrence at 9.5 months post treatment, with pT4b disease. The 12‐month cystectomy rate of evaluable patients was 3.3% (95% CI −3.1 to 9.8).

**Fig. 3:**
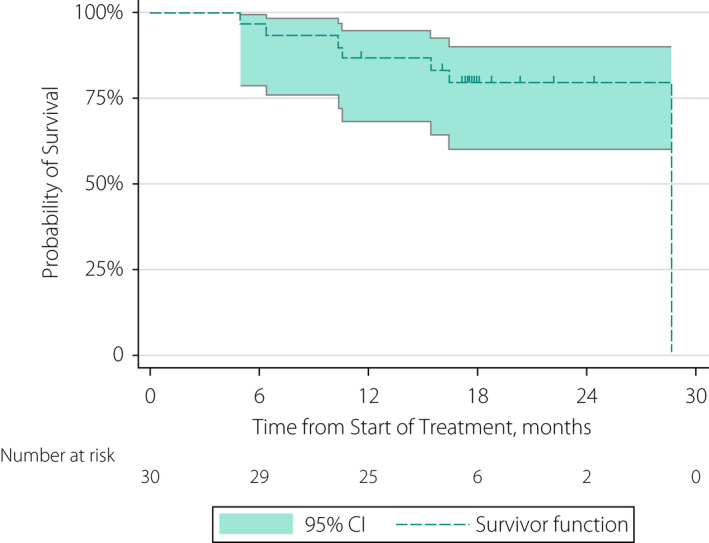
Overall survival. The secondary outcome of overall survival was defined as the interval in whole days between the start date of treatment and the date of death from any cause. Patients who did not die during the course of the trial were censored at the date of their last available assessment. Censored patients are indicated by short vertical lines. CI, confidence interval.

The overall survival rates at 6 and 12 months post treatment were 97% (95% CI 79–100) and 87% (95% CI 68–95), respectively (Fig. 3). Causes of death were bladder cancer‐related (four patients), aspiration pneumonia (one patient), atrial fibrillation/bronchiectasis (one patient), and treatment‐related (one patient). This treatment‐related death was attributable to cetuximab, as noted in Table 3.

Only 12 patients completed all six QoL questionnaires. Results from the EORTC QLQ‐C30 questionnaire demonstrated a dip in global health status at 1 month post treatment, which recovered by Month 3 (Appendix S3). This was consistent on all functional and symptom scales, with a concomitant increase in symptom scores. Data from the EORTC QLQ‐BLM30 demonstrated an increase in urinary symptoms at 1 month post treatment, which normalized by Month 3. All other items showed little change over time (Appendix S4).

## Discussion

The Phase I part of the TUXEDO trial confirmed feasibility and safety for the combination of radiotherapy with cetuximab and MMC/5FU chemotherapy. Recruitment was halted due to slow accrual during Phase II, resulting in the prespecified target of 45 patients not being reached. Recruitment was hampered by a separate competing national trial.

An exploratory analysis of Phase II, with limited power, was performed on data from the 30 evaluable patients recruited. Combined toxicity data and high dose intensity achieved for the chemoradiotherapy administered during TUXEDO demonstrated little additional toxicity from the addition of cetuximab when compared to published BC2001 trial data [10]. This was achieved without compromise in patient QoL and the results are in line with outcomes from BC2001 [9]. However, only approximately 33% of BC2001 patients also received neoadjuvant chemotherapy (compared to 79% in TUXEDO), with broadly similar results to the main trial, adjusting for case mix [25].

Very low rates of bladder recurrence were observed during TUXEDO, with 12‐month freedom from muscle invasion of 93% (95% CI 75–98) at 2 years; the comparable figure BC2001 was 82%. Although underpowered, the rates observed were similar, if not higher, than those observed in BC2001 with a similar case mix [10, 26]. Similar results were also observed with overall and metastasis‐free survival as well as freedom from cystectomy. Overall, it seems likely that this four‐component therapy tested is at least as effective as the BC2001 three‐component therapy, however, it is impossible to assess whether there may be a benefit compared to MMC/5FU alone due to the single arm, non‐randomized design of TUXEDO. Although cetuximab has limited single agent activity in urothelial cancer (which also applies to 5FU and MMC [27]), the benefit here is likely to derive from radio‐sensitization. The limited toxicity penalty makes the agent potentially combinable with other more recent approaches, such as immune checkpoint inhibition, which are being explored by ourselves (RadIO trial [28]) and others (KEYNOTE 992 trial [29]).

Prespecified within the trial design was an evaluation regarding continuing investigation in a randomized setting with cetuximab, and the safely and effectively delivered chemoradiotherapy, if the 3‐month disease control was within 60%–80%. The reported rate in TUXEDO was 77% (95% CI 58–90). Although the sample size target was not achieved and hence the CIs are larger than intended, and results should be interpreted with caution, these findings do suggest further evaluation of cetuximab in this setting would be worthwhile. Further translational work on tissue and urine collected during the trial is planned to assess possible biomarker‐based approaches, particularly given the evidence for such biomarkers in other cancers, such as colorectal cancers [30].

In summary, the results of this study suggest cetuximab is safe to combine with the UK radical chemo‐radiotherapy regimen MMC/5FU and shows high pelvic control rates, with future randomized clinical trials potentially worthy of consideration.

## Disclosure of Interests

Nicholas D. James received grants from Cancer Research UK pertaining to this research. Merck Serono Ltd donated cetuximab free of charge for use in the trial. All other authors declare no competing interests.

**Abbreviations**
5FU: 5‐fluorouracil
AE: adverse event
CIS: carcinoma *in situ*
EGFR: epidermal growth factor receptor
EORTC: European Organization for Research and Treatment of Cancer
MIBC: muscle‐invasive bladder cancer
MMC: mitomycin C
QoL: quality of life
TURBT: transurethral resection of bladder tumour

## Supporting information


**Appendix S1.** TUXEDO investigators.

**Appendix S2.** Adverse events.

**Appendix S3.** EORTC QLQ‐C30 global health status.

**Appendix S4.** Quality of life.

## Data Availability

Participant data and the associated supporting documentation will be available within 6 months after the publication of this manuscript. Details of our data request process is available on the CRCTU website. Only scientifically sound proposals from appropriately qualified research groups will be considered for data sharing. The decision to release data will be made by the CRCTU Director's Committee, who will consider the scientific validity of the request, the qualifications and resources of the research group, the views of the Chief Investigator and the trial steering committee, consent arrangements, the practicality of anonymizing the requested data and contractual obligations. A data‐sharing agreement will cover the terms and conditions of the release of trial data and will include publication requirements, authorship and acknowledgements and obligations for the responsible use of data. An anonymized encrypted dataset will be transferred directly using a secure method and in accordance with the University of Birmingham's IT guidance on encryption of datasets.
