# Supplementary material for: TUXEDO: a phase I/II trial of cetuximab with chemoradiotherapy in muscle‐invasive bladder cancer
Source: BJU Int. 2022 Aug 16;131(1):63–72. doi: 10.1111/bju.15864 (PMC10087008; doi:10.1111/bju.15864)
Supplement: Supplementary file 1 — Appendix S1. TUXEDO investigators. [file BJU-131-63-s004.pdf]

Supplementary Appendix A: TUXEDO Investigators

The TUXEDO trial investigators include the following;

Safety Review Committee: Dr Jim Barber (Velindre Cancer Centre, Cardiff, United Kingdom).

Centre and investigators (accrual): Isabel Syndikus, Zafar Malik and Chinnamani Eswar (Clatterbridge Cancer Centre, Wirral, United Kingdom; 11), Stephen Mangar (Imperial College Healthcare NHS Trust, London, United Kingdom; 5), Julian Money-Kyrle (Royal Surrey County Hospital, Guilford, United Kingdom; 0), Anna Lydon (South Devon Healthcare NHS Foundation Trust, Torquay, United Kingdom; 1), Johannes Van Der Voet (South Tees Hospitals NHS Foundation Trust, Middlesbrough, United Kingdom; 2), Nicholas James, Anjali Zarkar and Dan Ford (University Hospitals Birmingham NHS Foundation Trust, Birmingham, United Kingdom; 14).
