# Supplementary material for: TUXEDO: a phase I/II trial of cetuximab with chemoradiotherapy in muscle‐invasive bladder cancer
Source: BJU Int. 2022 Aug 16;131(1):63–72. doi: 10.1111/bju.15864 (PMC10087008; doi:10.1111/bju.15864)
Supplement: Supplementary file 2 — Appendix S2. Adverse events. [file BJU-131-63-s001.pdf]

Supplementary Appendix B: Adverse Events

Clinical Category / Toxicity	Number of Patients Affected N = 373	Number of Occurrences N = 482	Related to Treatment N = 353	Grade $\geq$3 N = 34
**Blood and lymphatic system disorders**				
dizzy	1	1	1	0
nose bleed	1	1	1	0
anemia	9	10	9	0
febrile neutropenia	1	1	1	0
low protein levels	1	1	0	0
**Cardiac disorders**				
atrial fibrillation	2	2	1	1
chest pain - cardiac	1	1	1	0
palpitations	2	2	1	0
reduced T-waves on ECG	1	1	1	0
**Ear and labyrinth disorders**				
bilateral ear yellow discharge	1	1	1	0
**Eye disorders**				
blurred vision	1	1	0	0
cataract	1	1	0	0
dry eye	1	1	1	0
eye pain	2	2	2	0
eye strain	1	1	0	0
itchy eyes	1	1	1	0
red, sore and itchy eyes	1	1	1	0
sore eyes	1	1	1	0
**Gastrointestinal disorders**				
abdominal pain	2	2	1	0
anal hemorrhage	2	2	0	0
constipation	5	6	5	0
diarrhea	18	29	25	4
dry mouth	2	2	2	0
flatulence	2	2	1	0
hemorrhoids	1	1	0	0
indigestion	1	1	1	0
mouth ulcers	2	2	2	0
mucositis oral	4	6	6	0
nausea	12	14	14	0
oral pain	3	3	3	0
proctitis	1	1	1	0
rectal hemorrhage	3	3	2	0
sloughing inside upper gum area	1	1	1	0
stomatitis	1	2	2	0
tenesmus	1	1	1	0

Clinical Category<br>Toxicity	Number of Patients Affected<br>N = 373	Number of Occurrences<br>N = 482	Related to Treatment<br>N = 353	Grade ≥3<br>N = 34
vomiting	5	6	3	0
**General disorders and administration site conditions**				
edema limbs	2	2	0	0
fatigue	12	18	14	0
fever	3	5	4	2
flu like symptoms	1	1	1	0
groin swelling	1	1	1	0
infusion site extravasation	1	1	1	0
mucositis nose	1	1	1	0
pain	3	3	1	0
swelling at picc line site	1	1	0	0
**Infections and infestations**				
anorectal infection	1	1	1	0
chest infection	1	1	1	1
oral thrush	1	1	1	0
paronychia	1	1	1	0
picc line infection	1	1	0	0
sepsis	1	1	0	1
shingles	1	1	1	0
toe infection	1	2	2	0
upper respiratory infection	2	5	0	0
urinary tract infection	5	9	3	0
**Injury, poisoning and procedural complications**				
dermatitis radiation	1	1	1	0
laceration to forehead	1	1	0	0
**Investigations**				
ALT increased	2	2	2	0
AST increased	2	2	2	0
creatinine increased	3	3	2	0
GGT increased	1	1	1	0
LDH raised	1	1	0	0
low bilirubin	2	4	0	0
low globulin	1	1	0	0
lymphocyte count decreased	1	2	2	0
neutrophil count decreased	3	8	8	3
platelet count decreased	7	14	14	3
raised globulin	1	1	0	0
raised LDH	2	2	0	0
raised urea	3	5	1	0
white blood cell decreased	3	7	7	2
**Metabolism and nutrition disorders**				
anorexia	8	9	6	0
hypercalcemia	1	1	0	0

Clinical Category<br>Toxicity	Number of Patients Affected<br>N = 373	Number of Occurrences<br>N = 482	Related to Treatment<br>N = 353	Grade ≥3<br>N = 34
hyperkalemia	1	1	0	0
hypoalbuminemia	3	3	1	0
hypokalemia	4	8	8	1
hypomagnesemia	9	11	6	0
hyponatremia	4	6	2	1
hypophosphatemia	3	4	4	0
protein levels decreased	1	1	0	0
**Musculoskeletal and connective tissue disorders**				
arthralgia	1	1	0	0
arthritis	1	1	0	0
back pain	3	3	2	0
cramps in hands and feet	1	1	1	0
osteoporosis	1	2	0	0
pain in extremity	3	3	0	0
t8 spinal collapse	1	1	1	0
**Nervous system disorders**				
dizziness	2	3	3	0
dysgeusia	2	3	2	0
lethargy	2	2	2	0
paresthesia	1	1	1	0
peripheral sensory neuropathy	1	1	1	0
tremor	1	1	0	0
**Psychiatric disorders**				
confusion	1	1	0	0
depression	1	1	1	0
insomnia	1	1	1	0
**Renal and urinary disorders**				
bladder irritation	1	1	1	0
bladder pain	1	1	0	0
cystitis noninfective	2	2	2	0
dull pain in bladder	1	1	1	0
dysuria	4	9	5	0
hematuria	5	6	4	3
hesitancy	1	1	1	0
nocturia	3	4	2	0
polyuria	1	1	0	0
proteinuria	1	1	1	0
urinary frequency	6	6	4	0
urinary incontinence	1	1	0	0
urinary retention	4	5	3	1
urinary tract obstruction	1	1	0	0
urinary tract pain	2	2	1	0

Clinical Category<br>Toxicity	Number of Patients Affected<br>N = 373	Number of Occurrences<br>N = 482	Related to Treatment<br>N = 353	Grade $\geq$3<br>N = 34
urinary urgency	3	3	2	0
**Reproductive system and breast disorders**				
erectile dysfunction	1	1	1	0
vaginal pain	1	1	0	0
**Respiratory, thoracic and mediastinal disorders**				
allergic rhinitis	1	2	2	0
cough	6	6	2	0
diaphragm pain	1	1	1	0
dyspnea	7	11	6	1
epistaxis	7	7	6	0
hiccups	1	1	1	0
laryngeal inflammation	1	1	0	0
pleuritic pain	1	1	1	0
pneumonitis	1	1	1	1
reduced sputum from chest infection	1	1	0	0
sore throat	2	2	1	0
**Skin and subcutaneous tissue disorders**				
bilateral back of knee rash	1	1	1	0
cracks on fingers	1	1	1	0
dry skin	8	8	5	0
erythema multiforme	1	2	2	0
fingers tingling and dry cracked nails	1	1	1	0
general rash	1	1	1	1
groin rash	1	1	1	0
hands red, dry and sore	1	1	1	0
light rash left arm	1	1	0	0
nail changes	1	1	1	0
pain of skin	4	4	3	0
palmar-plantar erythrodysesthesia syndrome	5	5	5	0
pruritus	6	6	6	0
purpura	1	1	1	0
rash acneiform	13	21	20	2
rash maculo-papular	15	34	33	3
rash on inner left and right thighs	1	1	1	0
rash on left and right buttocks	1	1	1	0
red feet	1	1	1	0
right thumb cracked	1	1	1	0
scalp and body tightness	1	1	1	0
skin hypopigmentation	1	1	1	0
skin rash/reaction	1	1	1	0

Clinical Category / Toxicity	Number of Patients Affected N = 373	Number of Occurrences N = 482	Related to Treatment N = 353	Grade ≥3 N = 34
sore on right side groin	1	1	0	0
sore skin to face	1	1	1	0
tender feet/skin cracked	1	1	1	0
**Vascular disorders**				
flushing	1	1	1	0
hypotension	1	1	0	0
lymphedema	1	2	1	0
superficial thrombophlebitis	1	1	1	0
thromboembolic event	3	3	2	3

ALT: alanine aminotransferase increased, AST: aspartate aminotransferase, ECG: electrocardiogram, GGT: gamma-glutamyl transferase, LDH: lactate dehydrogenase
