# Supplementary figures and images for: TUXEDO: a phase I/II trial of cetuximab with chemoradiotherapy in muscle‐invasive bladder cancer
Source: BJU Int. 2022 Aug 16;131(1):63–72. doi: 10.1111/bju.15864 (PMC10087008; doi:10.1111/bju.15864)

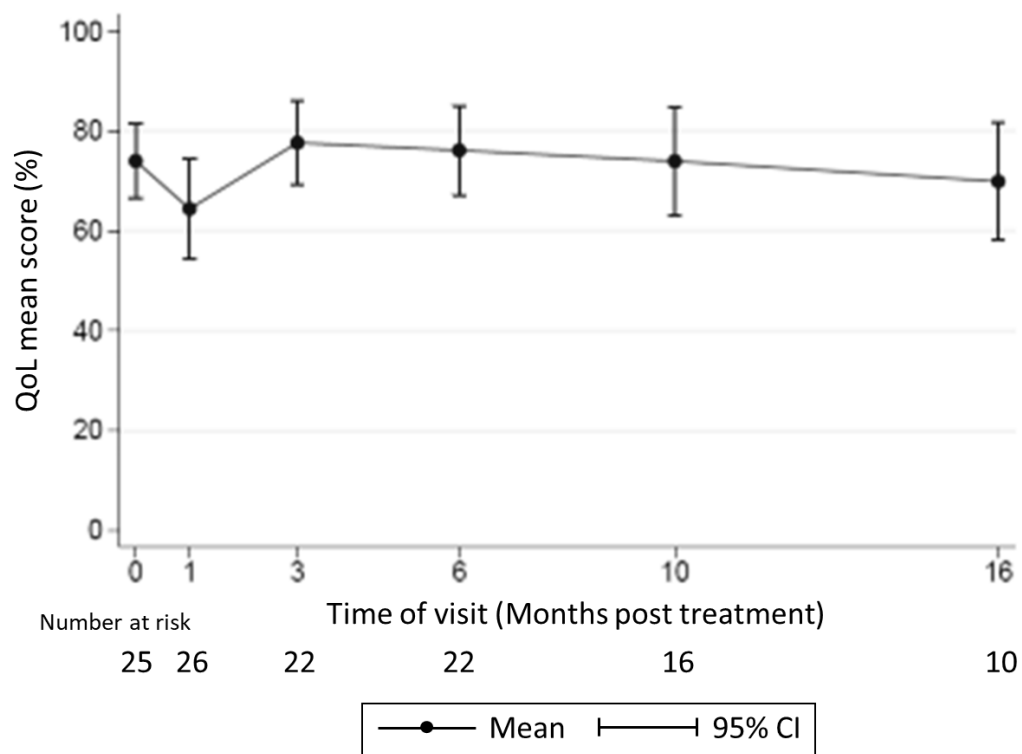

**Supplementary Appendix C. EORTC QLQ-C30 Global Health Status**

Supplement: Supplementary file 3 — Appendix S3. EORTC QLQ‐C30 global health status. [file BJU-131-63-s002.pdf]
