# Supplementary material for: TUXEDO: a phase I/II trial of cetuximab with chemoradiotherapy in muscle‐invasive bladder cancer
Source: BJU Int. 2022 Aug 16;131(1):63–72. doi: 10.1111/bju.15864 (PMC10087008; doi:10.1111/bju.15864)
Supplement: Supplementary file 4 — Appendix S4. Quality of life. [file BJU-131-63-s003.pdf]

Supplementary Appendix D: Quality of Life

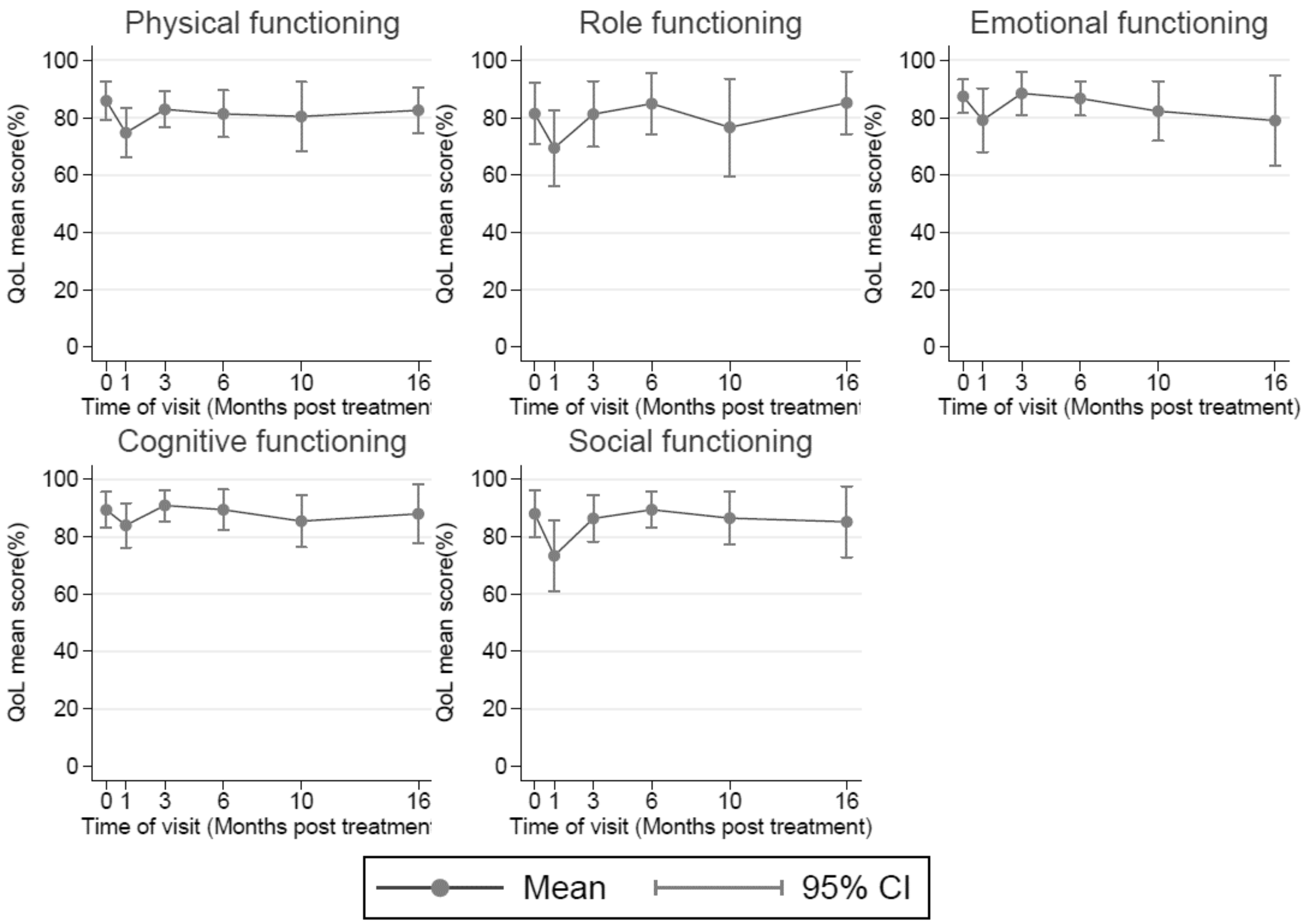

Figure D1. EORTC QLQ-C30 Functional Scales

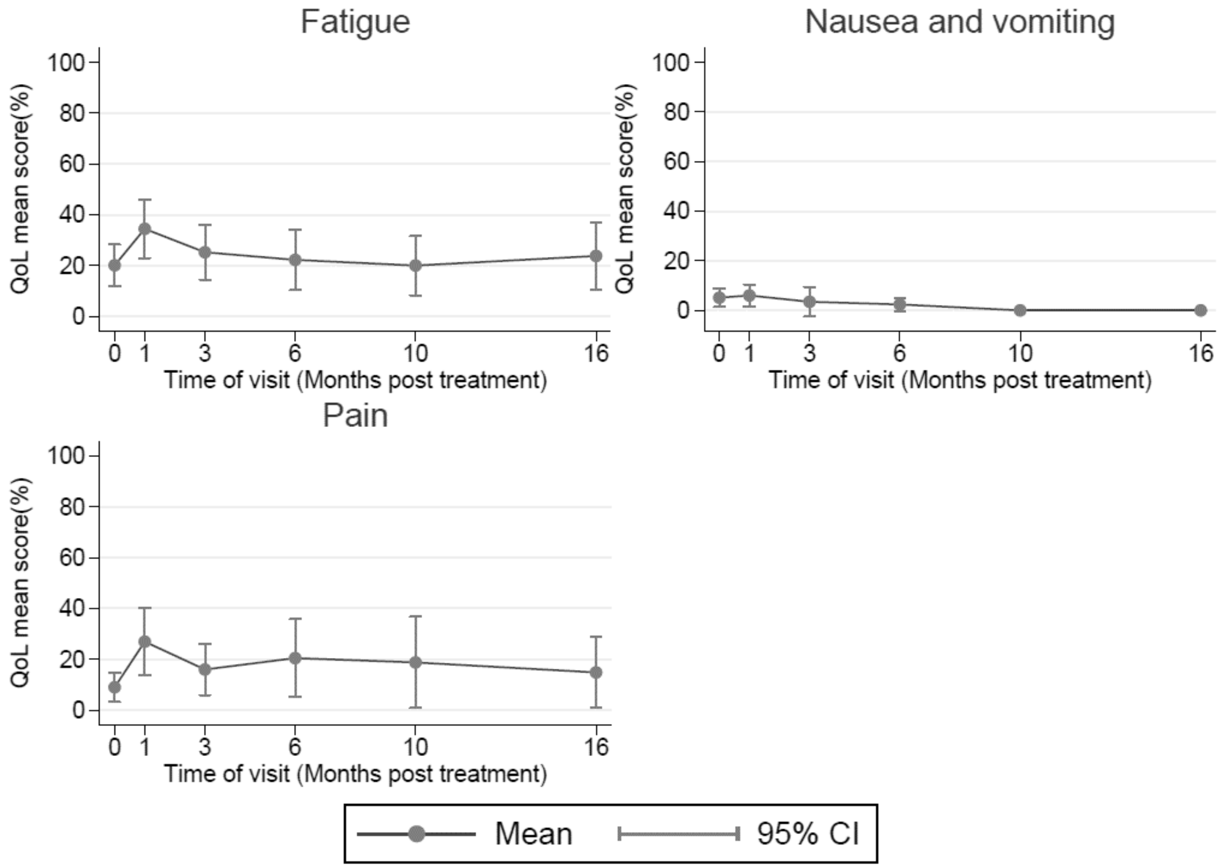

Figure D2. EORTC QLQ-C30 Symptom Scales

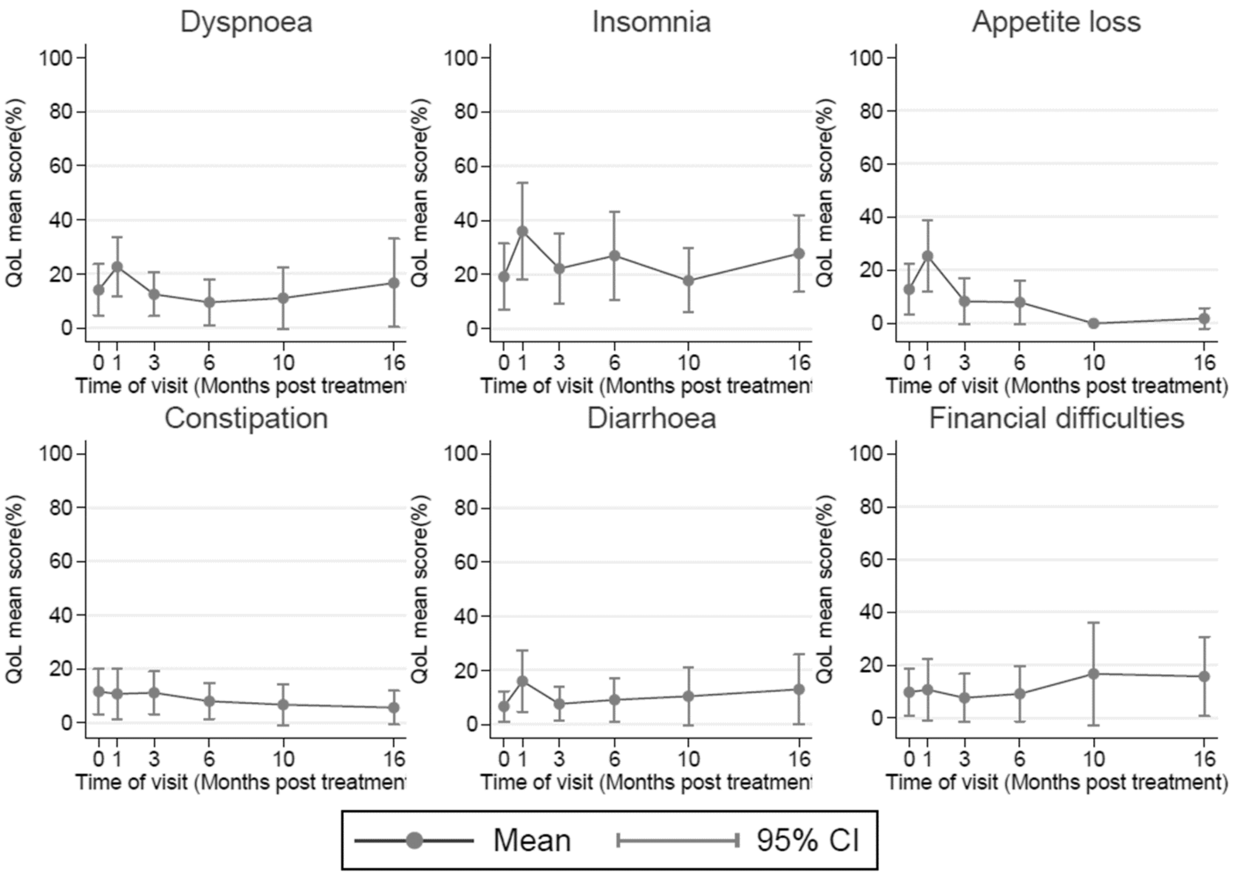

Figure D3. EORTC QLQ-C30 Symptom Items

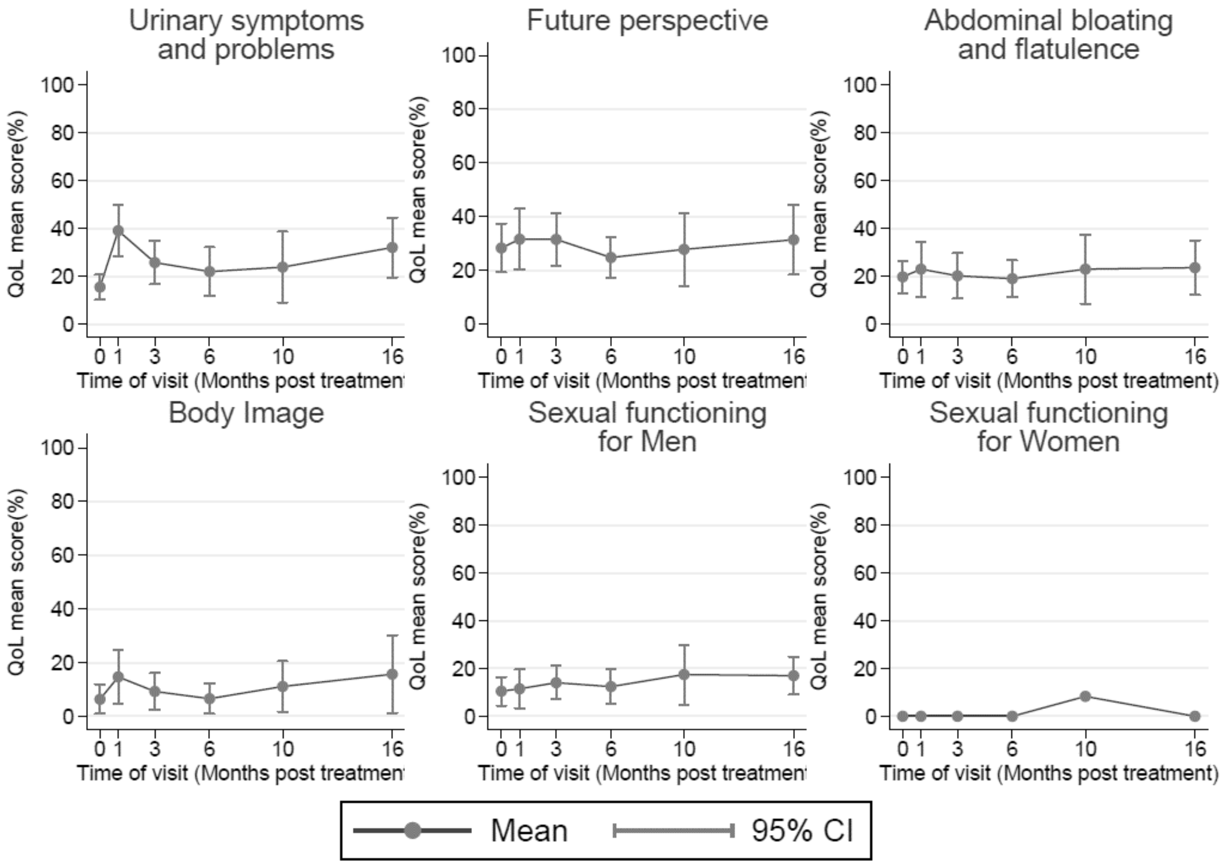

Figure D4. EORTC QLQ-BLM30 Items
